# Beyond anticoagulation: defining the role of left atrial appendage closure in stroke prevention for atrial fibrillation

**DOI:** 10.1093/europace/euag296

**Published:** 2026-11-06

**Authors:** A John Camm

**Affiliations:** Cardiovascular and Genomics Research Institute, School of Health & Medical Sciences, City St George’s University of London, Tooting Campus, Cranmer Terrace, London SW17 0RE, UK

**Keywords:** Atrial fibrillation, Stroke prevention, Direct oral anticoagulants, Left atrial appendage closure, Anticoagulation failure, Bleeding

## Abstract

Oral anticoagulation has a very strong evidence base for stroke prevention in patients with atrial fibrillation (AF) at elevated thromboembolic risk. Direct oral anticoagulants have improved the safety and convenience of treatment, yet important limitations remain, including residual ischaemic stroke, major bleeding, treatment discontinuation, and patient refusal. Left atrial appendage closure (LAAC) offers an alternative strategy for selected patients by excluding the principal anatomical source of AF-related cardiac thrombi. However, the appropriate role of LAAC remains controversial, particularly in patients who can and do tolerate anticoagulation. This viewpoint argues for a more individualized approach that distinguishes absolute contraindications from modifiable bleeding risks, treatment refusal or non-persistence, and stroke occurring despite adequate anticoagulation.

## Graphical Abstract

**Graphical Abstract:**
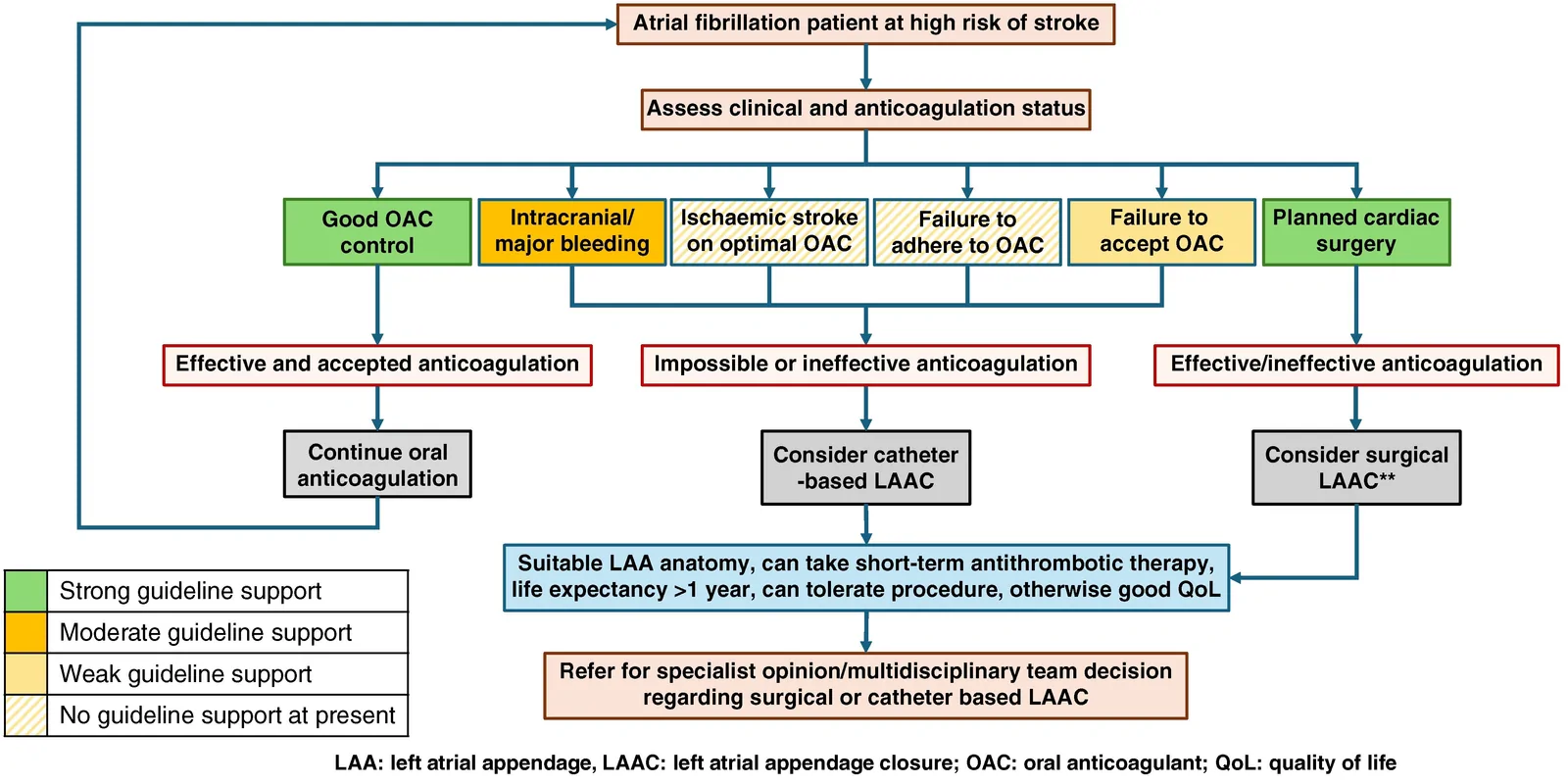

## The persistent burden of stroke in atrial fibrillation

Atrial fibrillation (AF) is a major risk factor for ischaemic stroke, and AF-associated strokes are often severe and disabling. Oral anticoagulation (OAC) substantially reduces this risk and remains the preferred preventive treatment for most eligible patients. Contemporary guidelines recommend anticoagulation according to individual thromboembolic risk, with direct oral anticoagulants (DOACs) generally preferred over vitamin K antagonists, except in specific circumstances, such as mechanical heart valves or moderate-to-severe mitral stenosis.^1,2^

Nevertheless, anticoagulation does not eliminate stroke risk. In major clinical trials, residual rates of ischaemic stroke or systemic embolism have generally been ∼1–2% per year, although rates vary with baseline risk, prior stroke, and treatment adherence. Major bleeding remains the major hazard associated with DOAC therapy; each year about 0.5% of patients sustain intra-cranial bleeding, and overall major bleeding occurs in 1.5–3.5%.^3^

## The limitations of long-term anticoagulation

Anticoagulants increase bleeding risk, including gastrointestinal (GI) bleeding and intracranial haemorrhage. Their effectiveness depends on adherence, appropriate dosing, renal function, and the management of drug interactions. Some patients discontinue treatment because of adverse effects, concerns about bleeding, inconvenience, cost, or personal preference.

It is essential to distinguish treatment refusal from treatment discontinuation and from a clinical contraindication. These groups have different implications for clinical practice and the potential role of left atrial appendage closure (LAAC). In the GARFIELD-AF registry, among 43 154 AF patients at high stroke risk, 30.8% were not anticoagulated. Among those in whom a reason for non-treatment was known, 12.5% (2.3% of the entire population) had refused anticoagulation.^4^

In the LAAC-REFUSAL study, 119 patients who refused OAC but accepted LAAC were compared to 238 controls and score-assessed stroke risk. The composite endpoint of cardiovascular death, stroke, or systemic embolism was significantly less than in those treated with LAAC (4.2% vs. 17.2% over 3 years), and technical success and bleeding were similar between group scores.^5^

Adherence to DOAC treatment relates to forgetfulness, lack of conviction that therapy is needed, inability to cope with mediation, inappropriate warnings from friends or relatives, etc. Educational help, best choice of medication, once vs. twice daily, etc. often significantly improved lack of adherence to therapy.^6^

Importantly, discontinuation of one anticoagulant does not necessarily mean abandonment of anticoagulation altogether: patients may switch drugs or temporarily interrupt treatment. Before considering a device-based strategy, clinicians should identify the underlying reason for non-persistence and address reversible barriers.

## Genuine contraindications: an important but imprecisely quantified population

A key consideration is the proportion of patients at elevated stroke risk who have a genuine contraindication to long-term OAC. Absolute, non-reversible contraindications are uncommon, but estimates of ∼1–5% are sometimes used to describe narrowly defined absolute contraindications.

Examples of potentially important contraindications include non-reversible intracranial bleeding and recurrent, severe bleeding from an uncorrectable source. Patients with AF and prior intracranial haemorrhage represent an important potential indication for LAAC, especially in those with amyloid angiopathy. The latest meta-analysis of 19 observational studies (1671 patients) found low procedural complication rates, with a 2.9% follow-up ischaemic stroke/TIA (transient ischaemic attack) rate and recurrent ICH 1.9%.^7^ These results suggest that LAAC is a better therapy than the standard care in patients with similar CHA_2_DS_2_-VASc (congestive heart failure, hypertension, age 75 years or older, diabetes mellitus, prior stroke or transient ischaemic attack, vascular disease, age 65-74 years, and sex category [female]) scores. However, evidence remains observational and requires randomized confirmation.

The PERSEO registry collected data on 70 of 1041 who underwent LAAC, at a median of 5 months post-discharge after a GI bleed. Left atrial appendage closure was associated with a lower risk (HR, 0.57; 95% CI, 0.37–0.89; *P* = 0.013) of recurrent composite events (stroke/transient ischaemic attack, systemic embolism, major or clinically relevant non-major bleeding, or death) or all-cause mortality (HR, 0.40; 95% CI, 0.22–0.72; *P* = 0.002).^8^

Advanced renal failure, particularly end-stage kidney disease requiring dialysis, represents a potentially important population for LAAC because of the competing risks of thromboembolism and anticoagulant-related bleeding. Left atrial appendage closure therapy may reduce the stroke risk, but bleeding is often higher when LAAC therapy is employed. Renal failure alone should not be regarded as an indication for LAAC unless anticoagulation has an unfavourable risk–benefit profile.

Conversely, previous bleeding, advanced age, frailty, falls, or a high bleeding-risk score do not automatically constitute an absolute contraindication. Many bleeding risks can be mitigated through blood pressure control, treatment of the bleeding source, adjustment of interacting medication, and selection of an appropriate anticoagulant dose.

## Stroke despite anticoagulation: identifying the mechanism

Breakthrough ischaemic stroke is an important clinical problem, but it should not automatically be interpreted as failure of the anticoagulant itself. In patients with AF who experience an ischaemic stroke while receiving anticoagulation, ∼25% of events were attributed to competing mechanisms, 30% to insufficient anticoagulation, and 45% to cardioembolism despite apparently sufficient anticoagulation.^9^

These findings illustrate why investigation is essential. Clinicians should assess adherence, dosing, renal function, and drug interactions. Evaluation should also consider large-artery atherosclerosis, small-vessel disease, and other potential causes of cerebral ischaemia.

For patients with a likely cardioembolic event despite adequate anticoagulation, LAAC is mechanistically attractive because it targets the source where most AF-related cardiac thrombi originate. Observational data suggest that LAAC therapy is highly effective in this group.^10^ Furthermore, LAAC cannot prevent strokes arising from every mechanism. Selecting patients according to the probable cause of their previous stroke is therefore crucial.

## Atrial fibrillation and significant stroke risk who is undergoing coronary artery bypass graft or valve surgery

There is strong evidence that surgical LAAC should be undertaken in patients with AF and CHA_2_DS_2_-VASc ≥ 2 who undergo cardiac surgery and continue OAC. LAAOS III randomized 4811 patients with AF and a CHA_2_DS_2_-VASc ≥ 2 who were undergoing cardiac surgery for another indication, predominantly coronary artery bypass graft (CABG) and/or valve surgery, to surgical LAA occlusion or no occlusion. Most patients (78%) continued OAC therapy. Stroke and systemic embolism were reduced from 7.0 to 4.8% (HR, 0.67; 95% CI, 0.53–0.85; *P* = 0.001) after a mean follow-up of 3.8 years.^11^

It is not yet fully resolved whether successful surgical LAA exclusion would be sufficient to allow some patients to discontinue long-term anticoagulation. However, guidelines are generally supportive of surgical LAAC irrespective of OAC continuation and strongly in favour of surgical LAAC as an adjunct to OAC therapy.

## Left atrial appendage closure: an alternative, not a universal replacement

Percutaneous LAAC provides a mechanical means of excluding the left atrial appendage from the systemic circulation. Randomized trials, including PROTECT AF, PREVAIL, and PRAGUE-17, have helped establish LAAC as a potential alternative to OAC in selected patients. PRAGUE-17, in particular, found LAAC non-inferior to DOAC therapy for a composite endpoint involving cardiovascular, neurological, and bleeding outcomes in a high-risk population. Most meta-analyses conclude that LAAC may not be as effective as OAC at preventing ischaemic stroke, but long-term bleeding is less and procedural complications are now relatively infrequent.^12^

Left atrial appendage closure is not superior to DOACs for every individual outcome or every patient group. The benefits must be weighed against procedural complications, including pericardial effusion, vascular injury, device-related thrombus, and peri-procedural stroke. Choosing LAAC thrombo-prophylaxis is also dependent on the clinical phenotype.

Left atrial appendage closure also does not eliminate antithrombotic treatment immediately. Most patients require a period of anticoagulation and/or antiplatelet therapy after implantation, together with follow-up imaging to assess device position, residual leak, and device-related thrombus. Patients unable to tolerate even short-term antithrombotic treatment may therefore present a particular challenge. The decision must also account for life expectancy, procedural suitability, and patient preference.

## Towards an individualized decision framework

A rational approach is to ask if the patient has a sufficiently high thromboembolic risk (assessed using validated risk scores) to warrant preventive treatment. If so, can and will the patient safely and reliably receive anticoagulation? If bleeding risk is modifiable, the preferred strategy is to address the underlying problem and optimize OAC rather than proceed directly to LAAC. If anticoagulation is unsuitable or problematic, LAAC may be appropriate depending on the reason for treatment intolerance, the mechanism of any previous stroke, and the feasibility of post-implant therapy.

Professional society guidelines, which rely predominantly on randomized control trial data, cautiously recommend LAAC therapy for those at most risk from DOAC failure or major bleeding. The deluge of observational data—often, but not always, largely ignored when guidelines are written—is more fully considered in guidance documents,^13^ suggesting a greater range of potential indications for LAAC therapy, but they serve mostly to generate hypotheses to be tested with more formal randomized studies^14^ (*Figure 1*).

**Figure 1:**
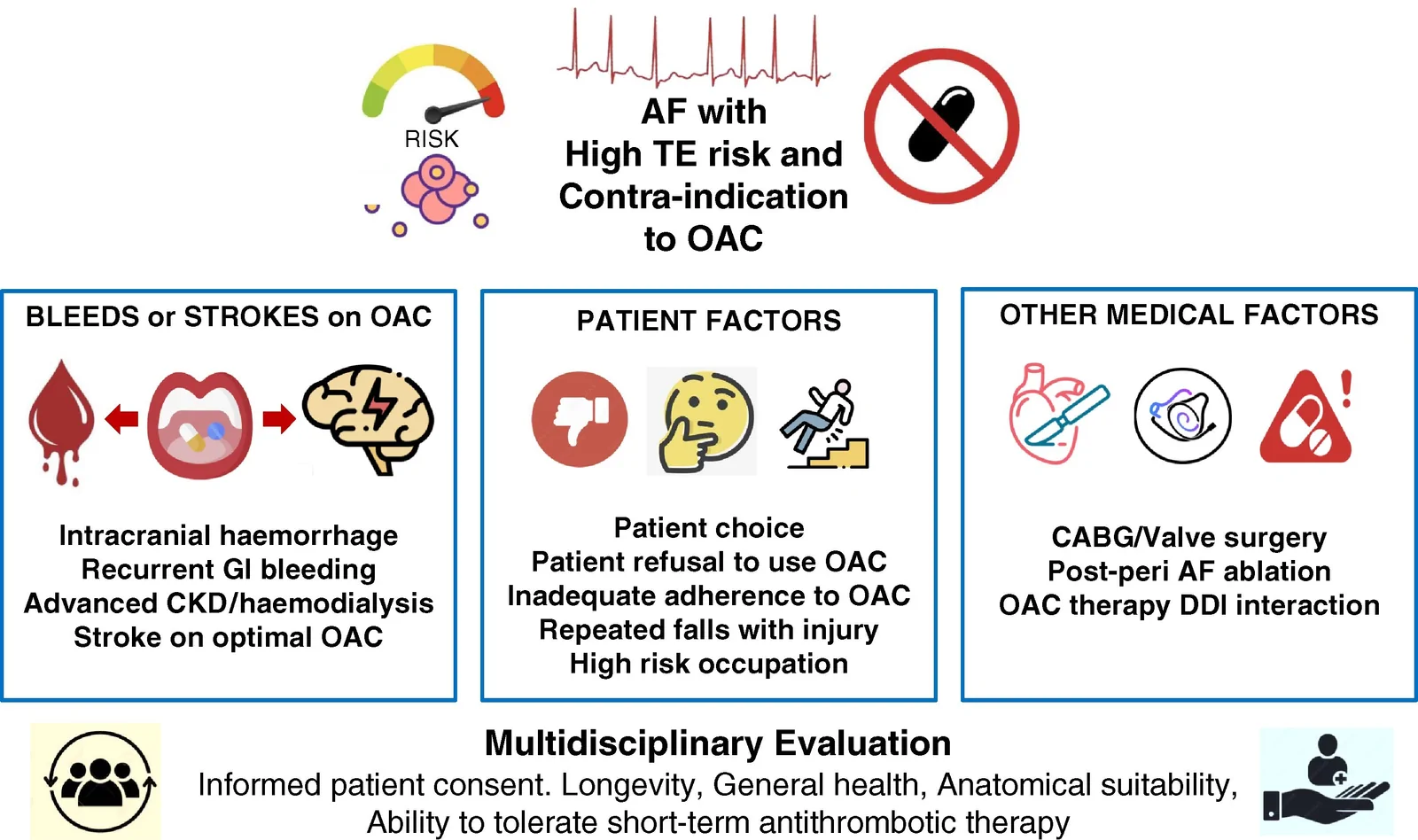
Patients who are unsuitable for oral anticoagulation may be candidates for left atrial appendage closure. AF, atrial fibrillation; CABG, coronary artery bypass graft; CKD, chronic kidney disease; DDI, drug–drug interaction; GI, gastrointestinal; OAC, oral anticoagulation; TE, thrombo-embolic.

The complexity of choosing LAAC as the most suitable therapy for any individual relies on shared decision-making. When making their choice, patients and physicians should understand the differences between lifelong medication with a significant bleeding risk and an invasive procedure with long-term benefits, but significant procedural risks.^15^

## Conclusion

Anticoagulation remains the foundation of stroke prevention in AF, but it is not a complete solution for every patient. Residual thromboembolism, bleeding, non-persistence, and genuine contraindications justify a more individualized approach. Left atrial appendage closure should be considered particularly for appropriately selected patients with a non-reversible contraindication to long-term OAC, or when OAC therapy is refused or not tolerated. Guideline recommendations remain very cautions, but guidance based on observational data suggests that more formal studies will demonstrate the full value of LAAC therapy.
